# Symptom Burden, Treatment Goals, and Information Needs of Younger Women with Pelvic Organ Prolapse: A Content Analysis of ePAQ-Pelvic Floor Free-Text Responses

**DOI:** 10.3390/jcm14155231

**Published:** 2025-07-24

**Authors:** Georgina Forshall, Thomas J. Curtis, Ruth Athey, Rhys Turner-Moore, Stephen C. Radley, Georgina L. Jones

**Affiliations:** 1 Psychology Department, School of Humanities and Social Sciences, Leeds Beckett University, City Campus, Leeds LS1 3HE, UK; r.turner-moore@leedsbeckett.ac.uk (R.T.-M.); g.l.jones@leedsbeckett.ac.uk (G.L.J.); 2 Norfolk and Norwich University Hospitals, Colney Lane, Norwich, Norfolk NR4 7UY, UK; tom.curtis@nnuh.nhs.uk; 3 Sheffield Teaching Hospitals NHS Foundation Trust, Herries Road, Sheffield S5 7AU, UK; ruth.athey@nhs.net (R.A.); stephen.radley@nhs.net (S.C.R.)

**Keywords:** pelvic organ prolapse, pre-menopausal, PROMs, patient-reported outcome measures, pelvic floor disorders, treatment goals

## Abstract

**Background/Objectives**: Pelvic organ prolapse (POP) is a common condition that significantly impacts quality of life. Research has focused largely on older women, while experiences of younger women remain relatively underexplored despite challenges unique to this population. Informed by the biopsychosocial model of illness, this study aims to assess the symptom burden, treatment goals, and information needs of younger women complaining of prolapse by analyzing questionnaire responses from an existing electronic Personal Assessment Questionnaire—Pelvic Floor (ePAQ-PF) dataset. **Methods**: Mixed-methods content analysis was conducted using free-text data from an anonymized multi-site ePAQ-PF dataset of 5717 responses collected across eight UK NHS trusts (2018–2022). A quantitative, deductive approach was first used to identify younger women (≤50 years old) with self-reported prolapse. ePAQ-PF scores for younger women with prolapse were compared with those aged >50 years, using Mann–Whitney tests. Free-text response data were analyzed inductively to qualitatively explore younger women’s symptom burden, treatment goals, and information needs. **Results**: Of the 1473 women with prolapse identified, 399 were aged ≤50 years. ePAQ-PF scores of the younger cohort demonstrated significantly greater symptom severity and bother than those aged >50, particularly in bowel, prolapse, vaginal, body image, and sexual health domains (*p* < adjusted threshold). Qualitative analysis undertaken to understand women’s concerns and priorities produced five health-related themes (physical health; functionality; psychosocial and emotional wellbeing; reproductive and sexual health; and healthcare journeys) and a sixth intersecting theme representing information needs. **Conclusions**: The findings highlight the substantial symptom burden of younger women with prolapse, as well as treatment goals and information needs specific to this population. The development of age-specific resources is identified as a requirement to support this group.

## 1. Introduction

Pelvic organ prolapse (POP) is a common condition: global estimates suggest that 40–60% of women may experience some degree of prolapse during their lifetime [1]. The prevalence of symptomatic POP increases with age, in part related to hormonal changes associated with menopause that may contribute to reduced pelvic floor muscle strength [2,3]. Approximately 80% of symptomatic cases occur in women aged 50 years and older [4,5,6], and surgical intervention is most frequently undertaken in women between 60 and 69 years of age [7]. As a result, most quality of life (QoL) research has concentrated on this older population [8,9,10], while comparatively little is known about the experiences and needs of younger women, who may face distinct challenges related to their pelvic floor and reproductive health, as well as to daily functioning and psychosocial wellbeing. This study seeks to address this gap by examining the symptom burden, treatment goals, and information needs of younger women with POP, analyzing responses from an existing electronic Personal Assessment Questionnaire—Pelvic Floor (ePAQ-PF) dataset. Details on ePAQ-PF, including its content and application, are provided in the Materials and Methods section.

Although benign, POP is a chronic condition that can substantially affect quality of life [7,11,12,13,14]. Symptoms such as vaginal bulge, pelvic pressure, and heaviness [5,15] may be exacerbated by physical activity [6,16], potentially resulting in activity limitation or avoidance, and reduced independence [17]. Urinary and bowel dysfunction are frequently reported and may interfere with activities of daily living, including caregiving, household responsibilities, and employment [6,18,19,20,21,22]. Additionally, many women report negative impact on emotional wellbeing, body image, and identity [12,18,20,22,23,24], compounded by the stigma associated with gynecological health conditions [6,12,14,19,25]. Reports of adverse impact on sexual function and intimate relationships are also common [14,16,18,19,22,26].

Emerging evidence suggests that some aspects of younger women’s experiences are distinct from those of women who develop prolapse later in life [6,14,18,27,28]. Pregnancy and childbirth, especially operative vaginal delivery, are well-established risk factors for prolapse [29,30,31], making the postpartum and reproductive years a critical period for symptom onset and progression. Studies that focus on younger women with POP have identified several population-specific challenges, including difficulties in managing POP symptoms alongside the demands of parenting, the impact of cyclical symptom fluctuation, concerns about future fertility and pregnancy, and limited access to postpartum care and information [6,14,18,19,27,28]. Some studies also suggest that younger women experience greater subjective symptom distress than older women, despite anatomic severity increasing with age [27,28]. Sociocultural expectations around femininity, motherhood, and physical fitness may contribute to this heightened burden [6,18].

Treatment options for POP include pelvic floor muscle training, lifestyle modification, vaginal pessaries, hormonal therapies, and surgery [32]. However, there are no specific guidelines tailored to the management of POP in younger women [33]. Evidence regarding treatment outcomes in this group remains limited, and surgical options may be constrained by concerns regarding the preservation of fertility and sexual function [33,34,35,36,37]. Younger age is also associated with increased surgical reoperation rates and adverse outcomes, further influencing shared decision-making [33,34,37,38,39].

Understanding the treatment goals of this population is therefore particularly important. Existing research indicates that the treatment goals of women with pelvic floor disorders are personal and highly subjective, often focusing on symptom relief, physical activity, emotional wellbeing, body image, relationships, and sexual function [40,41,42,43,44,45,46]. Goal attainment in urogynecology has been shown to correlate more strongly with patient satisfaction than objective clinical outcomes [42], and it has been suggested that goal-oriented treatment consultations improve shared decision-making [46]. However, there is a lack of research exploring the treatment goals of younger women with POP and the extent to which these goals may be informed by the specific symptom and QoL experiences in this population, as well as potential concerns around fertility and family planning.

Empowering women to participate in shared decision-making demands access to appropriate, timely, and relevant health information [47]. However, many younger women with POP report inadequate information about their condition, treatment options, and prognosis [6,18,48]. Resources provided by the UK National Health Service (NHS) [15,49,50,51] currently offer limited guidance on issues relevant to this population, such as menstruation, fertility, pregnancy and birth experiences, recovery timelines, and appropriate physical activity levels. These gaps may delay help-seeking [52], undermine informed consent, and increase psychological distress [8]. In accordance with the goals of the Women’s Health Strategy for England [53], which seek to improve women’s access to health information, understanding the specific information needs of younger women is vital, including when guiding the development of evidence-based resources to support symptom management and informed treatment decision-making.

### Aim of the Study

Despite the high prevalence and burden of POP, the experiences and needs of younger women remain relatively underexplored. These needs are likely influenced by age-specific factors, including fertility, parenting responsibilities, and gendered expectations of health and functionality.

This study aims to characterize the symptom burden, treatment goals, and information needs of younger women complaining of POP. ‘Younger’ women is defined here as aged ≤50 years, as this age range typically corresponds to the reproductive years [54,55]. This classification is clinically relevant due to the influence of reproductive status on the development and management of pelvic floor disorders.

To provide a comprehensive and context-sensitive understanding of women’s experiences, the study was guided by the biopsychosocial model [56,57,58] and Cohen’s Framework for Women’s Health [59], both of which recognize the complex interplay of biological, psychological, social, and structural determinants of women’s health.

## 2. Materials and Methods

### 2.1. Study Design

This study analyzed data from an existing database containing responses to the electronic Personal Assessment Questionnaire for the Pelvic Floor (ePAQ-PF). A mixed-methods approach (quantitative and qualitative) was used, combining content analysis of free-text responses with statistical analysis of questionnaire scores. Before describing how the sample for this study was obtained and the detailed methods of data analysis, an overview of the original dataset and analytic approach are provided below.

### 2.2. Summary of the Data and Analytic Approach

ePAQ-PF is a web-based patient-reported outcome measure (PROM) that provides a comprehensive assessment of self-reported symptoms and quality of life impacts in women with pelvic floor disorders [60,61,62,63]. The reliability, validity, and functionality of ePAQ-PF have been confirmed in multiple studies [61,64,65,66,67]. Since its development, ePAQ-PF has been used in routine urogynecology care in over 20 UK NHS hospitals.

Multi-site ePAQ-PF data were collated to include 5717 questionnaires from the latest version of ePAQ-PF (Version 18) over the period 1st April 2018 to 1st April 2022. The dataset included responses from women who completed ePAQ-PF as part of their routine clinical care at Sheffield Teaching Hospitals, Saint Mary’s Hospital, Manchester, Birmingham Women’s Hospital, South Tees Hospitals, Epsom and St. Helier University Hospitals, Norfolk and Norwich University Hospitals, and Stockport NHS Foundation Trust. The dataset was anonymized and only included women who were over 16 years old and had consented to the confidential use of their responses for approved research. In this study, the term ‘woman’ is used to refer to individuals presumed to be female based on clinical presentation. We acknowledge that not all individuals who experience gynecological or pelvic floor health conditions identify as women.

**Figure 1:**
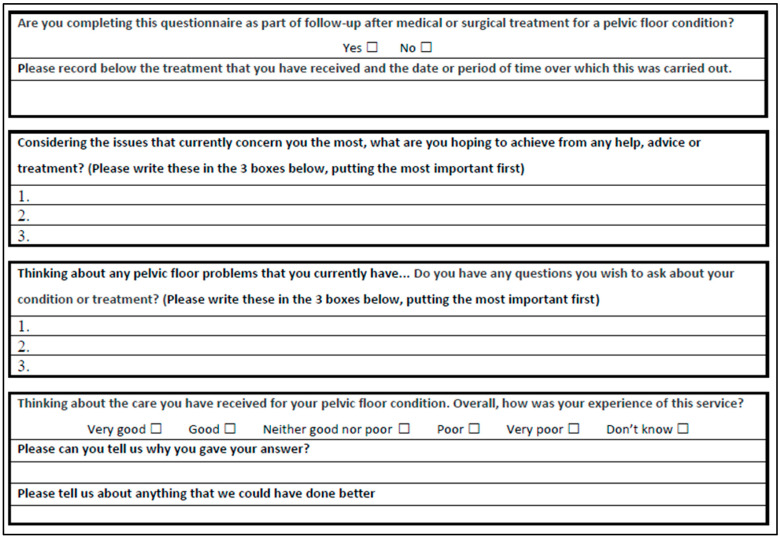
Open-ended questions (extracted from ePAQ-PF General Domain).

The core content of ePAQ-PF is structured in four dimensions relating to urinary, bowel, vaginal, and sexual symptoms. Each dimension comprises five symptom domains, which utilize standardized multiple-choice questions to assess the frequency and impact of pelvic floor symptoms. The urinary, bowel, and vaginal dimensions each include a QoL domain. An algorithm is applied to the responses provided to produce symptom severity, QoL, and impact (bother) scores. This provides a useful overview of the specific symptom areas that affect the individual the most. There is also a final ‘general’ dimension, in which women are invited to provide free-text responses to open-ended questions (Figure 1).

Content analysis was used to analyze the free-text responses. This systematic method of analysis can be used to derive valid inferences from various data sources—verbal, visual, or written—by identifying patterns and trends related to specific phenomena [68,69,70]. Its primary aim is to reduce large volumes of data into a structured and concise summary of key findings [71,72,73,74].

The analysis was conducted in two phases: first, to identify a sample of younger women (≤50 years old) with prolapse; and second, to examine their symptom burden, treatment goals, and information needs.

Both ePAQ-PF scores and free-text data were included in the analysis. Statistical data were analyzed using SPSS (version 29.0.1.0 (IBM Corp., Armonk, NY, USA)). Free-text data were imported into Microsoft Excel for analysis.

### 2.3. Patient and Public Involvement (PPI)

Patient and Public Involvement (PPI) members with lived experience of POP were recruited to review the development of the coding framework and preliminary findings, providing feedback that refined the analysis and informed recommendations for future research, as well as suggested improvements to patient care and information provision.

### 2.4. Sampling

ePAQ-PF has been developed and used in clinical practice to collect data from women with a broad spectrum of pelvic floor disorders. Anonymized ePAQ-PF response data were not linked to clinical records providing information on women’s medical histories. Therefore, to identify a sample of women with POP, content analysis of the free-text responses was conducted to categorize self-reported conditions. This phase used a quantitative, deductive approach to content analysis [68,75]. Following familiarization with the data, the first author (GF) developed a categorization matrix using pre-defined terms relating to pelvic floor disorders and other gynecological conditions (e.g., pelvic organ prolapse, incontinence, endometriosis). This was pre-tested on a random 10% sample of the data. To assess the validity of the matrix, the results were reviewed following feedback from members of the research team with clinical expertise (SR and RA), and revisions were made where necessary.

To test the reliability of the matrix, a further random 10% sample was independently coded by two members of the research team (GF and RA). Inter-coder reliability was calculated at 85.2%, using percentage agreement. Coding relating to prolapse categories (e.g., ‘prolapse only’; ‘prolapse and mesh complications’) was consistent, but there were some ambiguities relating to other condition categories (e.g., ‘birth-related perineal trauma’), which were resolved by discussion, and minor amendments were made to the matrix where necessary. The final matrix was then applied to the whole dataset (GF). This resulted in the identification of 1473 women with self-reported POP. This number included women reporting prolapse only, as well as women reporting prolapse concurrent to other conditions (e.g., incontinence, mesh complications). The dataset was then filtered by age to identify ‘younger’ women (aged ≤ 50 years).

### 2.5. Analyzing Symptom Impacts, Treatment Goals, and Information Needs

The second phase of content analysis employed a qualitative, inductive approach to content analysis [73,76,77,78] to identify the concerns of younger women complaining of POP, with respect to their symptoms and quality of life, their priorities for treatment, and their information needs.

The first coder (GF) read through the data several times and made observational notes on initial impressions, recurring topics, and significant statements. Open coding was then undertaken, which involved condensing the text from a random 20% sub-sample of the data into meaning units (i.e., a brief description that conveyed a single idea or concept). The condensed meaning units were assigned initial descriptive labels (codes) that were rooted in the data but conceptualized with the research aims in mind [73].

The coding scheme was developed by reviewing and revising these initial codes, which were compared for similarities and differences and abstracted into categories. The latent content of the categories was then generated into themes and sub-themes relating to reported symptom burden, treatment goals, and information needs. To accompany the coding scheme, a codebook was created to index each code, including the code name, definition, inclusion and exclusion criteria, and exemplar quotes.

To assess validity, the coding scheme was reviewed by clinical members of the research team (SR and TC) and the PPI panel, with reference to an anonymized random 10% sample of the data. Through a process of feedback, reflection, and discussion, the coding scheme and codebook were amended as necessary. This included introducing new codes where appropriate (e.g., reassigning nausea and bloating from ‘pain and discomfort’ to a newly created code ‘gastro-intestinal symptoms’) and re-conceptualizing some of the categories and themes (e.g., an initial theme ‘relationships and social life’ became ‘psychosocial and emotional wellbeing’).

A second coder (TC) was then trained to use the revised coding scheme and codebook. The first and second coders independently coded a random 10% sample of the data. Inter-coder reliability was calculated using percentage agreement at 81%. To finalize the coding scheme, GF and TC discussed discrepancies in coding and made some minor amendments to improve clarity and usability, and the codebook was refined and adjusted. To establish intra-rater reliability, the first coder (GF) coded a further random 10% sample on two separate days, one week apart. This was calculated using percentage agreement, equaling 94%. The final coding scheme was then applied to the whole sample by the first coder (GF). While doing so, notes were made on frequently mentioned concerns, and exemplar quotes relating to specific themes were collated. Quotes reported in the results are the most representative for each conceptual area; small edits were made to correct spelling and punctuation.

### 2.6. Statistical Analysis

In addition to qualitatively analyzing the symptom burden of POP in younger women (≤50 years), symptom severity and impact were assessed by comparing the ePAQ-PF symptom, QOL, and impact domain scores of this group with those of older women (>50 years). Shapiro-Wilk tests indicated that all scores were not normally distributed (*p* < 0.001), so non-parametric comparisons were conducted, using Mann–Whitney tests. Given the number of scores to compare (20 symptom and QoL domain scores and 17 impact scores), Bonferroni’s correction was applied to control for Type 1 errors. The adjusted significance threshold was set at *p* < 0.0025 when comparing symptom and QoL domain scores, and *p* < 0.0029 when comparing impact scores. Effect sizes (*r*) were calculated using the formula $r=\frac{z}{\sqrt{N}}$, appropriate for Mann–Whitney tests, to aid interpretation of the magnitude of group differences.

### 2.7. Researcher Reflexivity

This study was conducted by a multidisciplinary team of health and social psychologists (GF, GJ, RTM) and urogynecologists (SR, RA, TC), combining expertise in patient-experience research, qualitative methods, and clinical management of pelvic floor disorders. The psychology team’s focus on the biopsychosocial model of health informed the study’s thematic framework, while the clinicians ensured clinical relevance and contextual accuracy.

While the dataset was fully anonymized and researchers had no direct contact with respondents, we recognize that our disciplinary perspectives and assumptions may have influenced the research design, analysis, and interpretation, though we endeavored to keep the principles of patient-centered care, quality of life, and shared decision-making central to the research. Reflexivity was maintained through ongoing team discussion, collaborative development of the coding framework, and review by a PPI panel with lived experience, strengthening the rigor and trustworthiness of the findings.

This study adheres to the EQUATOR Standards for Reporting Qualitative Research (SRQR) [79].

## 3. Results

Of the 1473 women self-reporting prolapse identified in the first phase of content analysis, 399 were aged ≤50 years at the time of completing the questionnaire; 1074 were aged >50 years. The demographic characteristics of these two groups are provided in Table 1.

### 3.1. Severity and Impact of Pelvic Floor Symptoms

#### 3.1.1. Symptom and Quality of Life Domain Scores

An algorithm is used to calculate ePAQ-PF symptom and quality of life domain scores, transforming responses into a 0–100 scale, where 0 indicates the best and 100 the worst health status. When comparing the two age groups, statistically significant differences in scores were observed in all symptom and QoL domains, except ‘Pain and Sensation—Urinary’, ‘Voiding—Urinary’, ‘Continence—Bowel’, and ‘Capacity—Vagina’ (Table 2). Women aged ≤50 years had higher scores in all significantly different domains, except the ‘Overactive Bladder’ domain, where women aged >50 years scored higher. These findings suggest that the younger cohort of women experienced a greater burden of pelvic floor symptoms, particularly in relation to overall bowel function, prolapse, and other vaginal symptoms, body image, and symptom-related quality of life. Women aged ≤50 years also reported significantly worse sexual wellbeing. For example, the ‘General Sex Life’ median score of 50.00 (compared with 16.67 in women aged >50) and the wide interquartile range indicate considerable distress in this domain. Effect sizes (r) support the clinical relevance of the findings, with the largest effects observed in domains related to sexual function and body image.

#### 3.1.2. Impact Scores

Impact scores, which measure the degree of symptom-related bother on a 4-point Likert scale (0 = ‘not a problem’ to 3 = ‘a serious problem’), revealed significant differences between age groups across most domains (Table 3). Women aged ≤50 years attributed significantly greater symptom-related impact than those aged >50 years in most areas. Notably, younger women attributed significantly higher impact to vaginal pain and sensation, prolapse symptoms, and sexual health issues, including dyspareunia, reduced general sexual satisfaction, and sex-related urinary, bowel, and vaginal symptoms. Similarly, younger women had higher median scores for ‘Stress Urinary Incontinence’, ‘Constipation’, ‘Evacuation—Bowel’, and ‘Body Image’. Although median scores for the ‘Irritable Bowel’ were identical for both groups, women aged ≤50 years had significantly higher rank distributions (*p* < 0.001), indicating greater overall symptom burden. No statistically significant differences were observed in the domains of ‘Pain and Sensation—Urinary’, ‘Voiding-Urinary’, ‘Overactive Bladder’, or ‘Continence—Bowel’, indicating comparable levels of symptom bother in these areas across age groups. The largest effect sizes (*r*) were again found in the body image and sexual function domains, highlighting the substantial impact of these issues on younger women.

### 3.2. Symptom Burden, Treatment Goals, and Information Needs of Younger Women

**Figure 2:**
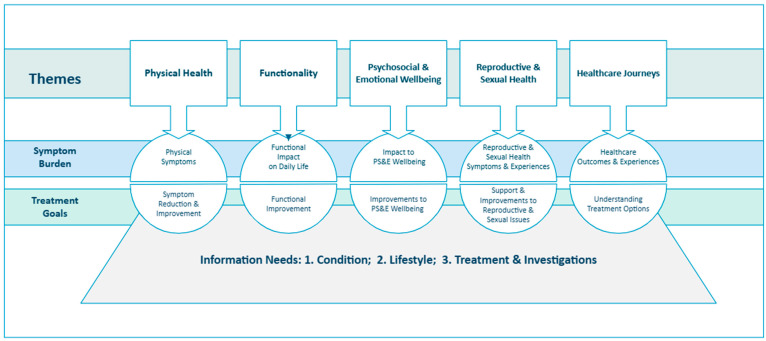
Thematic framework representing symptom burden, treatment goals, and information needs of younger women complaining of prolapse.

Qualitative content analysis of free-text responses from women aged ≤50 years self-reporting prolapse (n = 399) identified five health-related themes and ten sub-themes, as shown in Figure 2. Each theme captures women’s concerns about symptom burden and its impact on quality of life, as well as their treatment goals. An additional theme, reflecting younger women’s information needs, intersects with all five health-related themes and comprises three sub-themes. Appendix A (Table A1, Table A2 and Table A3) provides synopses of the key issues related to symptom burden, treatment goals, and information needs, respectively, each accompanied by the most representative illustrative quotes.

#### 3.2.1. Theme 1: Physical Health

The theme Physical Health includes the sub-theme, Physical Symptoms, capturing women’s embodied, lived experience of prolapse-related symptoms, as well as the sub-theme, Symptom Reduction and Improvement, relating to their priorities in reducing the burden of these symptoms and impact on overall physical health.

1. Symptom Burden: Physical Symptoms

Women described symptoms specific to prolapse as well as associated symptoms, such as urinary and bowel dysfunction, gastrointestinal symptoms (e.g., bloating), and pain and discomfort. They also disclosed other vaginal symptoms and gynecological disorders (e.g., cystic ovaries, fibroids).

*‘Vaginal prolapse and dragging heaviness in abdomen’*

*(age 39)*

*‘Wind and bowel urgency and incontinence. Bladder incontinence’*

*(age 43)*

Women also commonly referred to additional and compounding burdens that related to their primary prolapse symptoms:

*‘Bowel problems, having to keep taking laxatives as I have been doing for many years’*

*(age 48)*

*‘The pain makes me feel sick often’*

*(age 39)*

2. Treatment Goals: Symptom Reduction and Improvement

Women reported treatment goals that closely aligned with their subjective symptom experiences. These related mainly to alleviating specific symptoms, particularly in relation to toilet issues, such as urinary leakage and difficulty emptying the bowel, as well as reducing pain and discomfort. It was also common for women to seek improved health outcomes through a repair or removal of the prolapse itself.

*‘Get rid of prolapse so that I can open my bowels without having to support the bowel vaginal wall’*

*(age 45)*

#### 3.2.2. Theme 2: Functionality

The theme Functionality comprises two sub-themes: the first captures the impact of prolapse on women’s ability to engage in everyday activities; the second reflects their desire to restore or enhance their functional capacity.

1. Symptom Burden: Functional Impact on Daily Life

As a result of their symptoms, women reported significant restrictions in role functioning and in their ability to perform daily activities. These disruptions extended across employment, caregiving responsibilities, and household tasks, highlighting the condition’s interference with both physical capability and social roles. In particular, the inability to engage in exercise, due to pain, discomfort, reduced mobility, or fear of symptom progression, emerged as a recurring concern, reflecting the physical constraints imposed by prolapse.

*‘I kept having excruciating pain in my tummy and vaginal area. I took myself to hospital and a gynecologist said I had a prolapse of the womb, as well as loose skin which hangs down, causing more pain/infections/embarrassment and [difficulties in] the enjoyment of a sex life/social activities/day to day housework/exercise and anything which involves moving around’*

*(age 33)*

2. Treatment Goals: Functional Improvement

Women frequently reported treatment aims related to restoring everyday function, including the ability to walk without pain, return to exercise, and carry out daily tasks without discomfort or anxiety. These priorities reflect the impact of prolapse on mobility and daily living, and the importance placed on regaining physical confidence and independence.

*‘Help returning to a normal life, i.e., […] walking without pain’*

*(age 28)*

*‘To find a pessary that works so I can work out again and lift children while keeping pressure off pelvic floor’*

*(age 38)*

#### 3.2.3. Theme 3: Psychosocial and Emotional Wellbeing

The theme Psychosocial and Emotional Wellbeing comprises two sub-themes: one addressing the emotional, psychological, and social impact of living with prolapse, and another reflecting women’s intentions to improve their wellbeing across these interconnected areas.

1. Symptom Burden: Impact on Psychosocial and Emotional Wellbeing

POP was frequently described as impacting multiple aspects of social and relational life, including social engagement, intimate relationships, and participation in family or parenting activities. Many women reported significant effects on their mental health, citing feelings of depression, anxiety, and emotional distress. Prolapse also disrupted their sense of self, with negative impacts on self-image and body confidence. For some, these experiences led to a diminished perception of their overall quality of life, underscoring the emotional burden of living with prolapse.

*‘I’m really depressed about my vagina and how it makes me feel.’*

*(age 23)*

*‘I have a toddler and 10-year-old, and I can’t even be the mum they deserve’*

*(age 32)*

2. Treatment Goals: Improvements to Psychosocial and Emotional Wellbeing

Women frequently identified treatment priorities that centered on restoring psychosocial wellbeing, particularly the ability to socialize, regain confidence, and improve emotional and psychological health. These goals reflected a desire to reclaim a sense of normalcy, reduce feelings of embarrassment or isolation, and reconnect with meaningful aspects of life, such as social activities, relationships, and self-worth.

*‘Correction of prolapse […] so I can enjoy dancing (nights out) and confidence, no longer being embarrassed […] stop taking antidepressants’*

*(age 42)*

*‘Be able to live a more normal life’*

*(age 28)*

#### 3.2.4. Theme 4: Reproductive and Sexual Health

The theme Reproductive and Sexual Health comprises two sub-themes: Reproductive and Sexual Health Symptoms and Experiences, which reflect women’s symptom-related difficulties in these areas, while Support and Improvements to Reproductive and Sexual Issues represents their treatment needs and expectations. These sub-themes span three key areas, addressed in the following sections below: sexual function and satisfaction; pregnancy and childbirth; and menstrual/cyclical health.

1. Symptom Burden: Reproductive and Sexual Health Symptoms and Experiences
  - Impact on Sexual Function and Satisfaction

Women described a range of issues affecting sexual function, including coital incontinence, dyspareunia, dysorgasmia, uncomfortable physical sensations (e.g., in relation to vaginal laxity), and reduced sexual sensitivity. Many also expressed dissatisfaction with their sex life, citing loss of libido, diminished confidence, avoidance of sexual activity, and concerns about forming new sexual relationships.

*‘This is serious for me, I can’t meet anyone or be with anyone, because sex is important and I want to have sex with someone and feel sexy, but I just can’t’*

*(age 23)*

*‘Have to empty my bowel prior to having sex for fear of leakage during sex’*

*(age 45)*

  ii. Pregnancy and Childbirth Concerns

Some women also reported ongoing concerns about physical damage sustained during pregnancy and childbirth, including perineal trauma, episiotomy scarring, and pelvic floor injury. Several associated the onset or worsening of prolapse symptoms with specific birth experiences, such as instrumental delivery or delivering large babies, while others expressed uncertainty about the extent of their injuries.

*‘After birth of 2nd child vagina opening is too wide due to stitches coming undone’*

*(age 39)*

*‘First daughter ventouse (now 9) and second daughter (now 5 years old), forceps which caused the problems’*

*(age 40)*

  iii. Menstrual/Cyclical Health Concerns

Several women described menstruation-related difficulties that were either directly impacted or exacerbated by their experience of prolapse. Common concerns included difficulty using menstrual products (e.g., tampon displacement), dysmenorrhea, heavy bleeding, and pronounced premenstrual symptoms. Some women also noted a perceived worsening of prolapse symptoms in relation to hormonal fluctuations during their menstrual cycle, such as increased pelvic heaviness or vaginal bulge in the premenstrual phase.

*‘Prolapse bulging, inability to keep tampon in’*

*(age 33)*

*‘My bladder issues are very related to my cycle. What holistically can be done?’*

*(age 41)*

2. Treatment Goals: Support and Improvements to Reproductive and Sexual Issues
  - Restoration of Sexual Function and Satisfaction

Women highlighted the restoration of sexual wellbeing as a key priority, with treatment goals focused on resuming an active and enjoyable sex life, reducing pain and discomfort during intercourse, and improving genital appearance to alleviate embarrassment and enhance confidence.

*‘Reduce pain/discomfort during sexual intercourse and regain more enjoyment of sex’*

*(age 36)*

*‘To be able to have a sex life without embarrassment by being tighter and looking normal externally’*

*(age 39)*

  ii. Support Relating to Reproductive Health

Concerns about previous pregnancy and birth experiences often coexisted with a perceived lack of follow-up care and a desire for further support, treatment, or surgical intervention.

*‘Since I have given birth, my condition has gotten worse, and I have not received any further treatment’*

*(age 26)*

*‘Relief from pain of episiotomy scar tissue. Possible refashioning which I was due to have [date]. Regain control over my pelvic floor’*

*(age 38)*

Other treatment-related preferences were often shaped by family planning intentions. Some women wished to delay or avoid surgical options due to the desire for future pregnancy, while others questioned eligibility for surgery in the absence of reproductive intentions. Across the responses, there was a clear emphasis on the importance of receiving treatment that was tailored to individual reproductive priorities.

*‘Do I have non-surgical options as considering one more baby at some point?’*

*(age 36)*

*‘I have no interest in having children biologically, therefore am I eligible for prolapse surgery?’*

*(age 28)*

  iii. Improved Menstruation Experiences

Treatment goals in this area centered on alleviating menstrual discomfort, improving the ability to manage menstruation effectively (e.g., using preferred period products), and minimizing prolapse symptom exacerbation linked to hormonal or cyclical changes. These goals demonstrate a need for holistic and tailored management that addresses the interplay between general, gynecological, and pelvic floor health.

*‘Strengthening pelvic floor to use a menstrual cup again’*

*(age 32)*

*‘Reduce heavy feeling in vagina when due a period.’*

*(age 47)*

#### 3.2.5. Theme 5: Healthcare Journeys

The theme, Healthcare Journeys, includes two sub-themes. The first, Healthcare Outcomes and Experiences, captures women’s perceptions of their experiences of care, as well as outcomes relating to prior treatments, ongoing management concerns, and expectations or anxieties regarding future care. The second sub-theme, Understanding Treatment Options, relates to women’s perspectives on treatment options, shaped by both prior experiences and anticipated needs.

1. Symptom Burden: Healthcare Outcomes and Experiences
  - Ongoing Treatment Concerns

Women frequently reflected on previous interventions, describing a range of outcomes that shaped their attitude towards care. While some expressed satisfaction, many reported unresolved symptoms or temporary benefits followed by symptom recurrence, and complications such as pain or mesh-related issues. Concerns around continued symptom burden following treatment also included unresolved bladder or bowel issues, the onset of dyspareunia and other sexual issues, and dependence on symptom management strategies (e.g., antibiotics, laxatives).

*‘Vaginal ring pessary inserted at appointment as moderate prolapse […] Really suffering with pelvic pain and back ache and anus pressure’*

*(age 35)*

*‘I had a Rectopexy […] I have now developed superficial dyspareunia to the extent that penetration is completely impossible’*

*(age 50)*

  ii. Future Treatment Concerns

Anticipatory concerns about future treatment were also prevalent. Women reported anxiety regarding potential complications and whether existing comorbidities or medications might affect treatment options. Avoidance of invasive procedures, particularly surgery, was a notable priority. Some also raised concerns about how treatment might impact sexual function, recovery, or long-term quality of life.

*‘Because I also have a slight rectocele, will my cystocele repair make the rectocele more of a problem?’*

*(age 49)*

*‘I am only 45 and I have concerns about non-surgical treatment that has to be repeated regularly’*

*(age 45)*

  iii. Perception of Care

Women’s encounters and experiences with clinicians significantly shaped their overall perception of care. Positive interactions, characterized by empathy, clear communication, and reassurance, helped mitigate feelings of anxiety, embarrassment, or vulnerability during appointments.

*‘I was very apprehensive before attending as it’s a very personal issue. Everyone I met was supportive and understanding and put me at my ease. I feel I received appropriate advice, and the proposed treatment plan is as I hoped.’*

*(age 27)*

Conversely, negative encounters had a compounding effect on women’s psychological burden. Experiences of feeling dismissed, not believed, or poorly supported were described as distressing. Organizational issues, such as delayed referrals, long waiting times, the impact of COVID-19 on service provision, and a lack of follow-up care, further exacerbated dissatisfaction and, in some cases, reduced trust in the healthcare system.

*‘My initial concerns post-op were dismissed, which resulted in emergency surgery. Supposed to have follow-up or response via PALS. Not had post-op meeting with head of gynecology’*

*(age 46)*

2. Treatment Goals: Understanding Treatment Options

This sub-theme captures women’s preferences, expectations, and concerns regarding the management options available for pelvic organ prolapse. Unlike treatment goals aimed directly at symptom relief or quality-of-life improvements, these responses focus on the perceived appropriateness, accessibility, and acceptability of specific interventions. Some participants expressed a desire for further treatment following unsatisfactory outcomes from prior interventions, while others sought clarification on what options might be available to them and how these might align with their individual needs and circumstances.

In terms of specific types of treatments, many women expressed a clear desire for surgical interventions, while others wished to avoid surgery in favor of more conservative options, including pessary fitting, hormone replacement therapy (HRT), and physiotherapy. In some cases, women discussed wanting to stop a particular type of treatment due to a perceived lack of efficacy or concerns about safety and side effects. These accounts demonstrate that women’s treatment goals are shaped not only by their current symptom burden but also by their previous healthcare experiences and evolving expectations of care.

*‘How can I go about trying other pessaries (ring pessary has not worked for me)’*

*(age 38)*

*‘I don’t want that mesh thing that causes horrible pain and infection!’*

*(age 49*)

#### 3.2.6. Theme 6: Information Needs

The need for accessible, relevant, and situation-specific information emerged as a central concern in women’s free-text responses. Women frequently requested advice to help them understand their diagnosis and manage their condition effectively. These information needs were not isolated from but were closely intertwined with, and often foundational to, the five other key themes discussed above. Women’s questions extended beyond the physical aspects of prolapse and symptom management to include concerns about how to live with the condition day-to-day, and how to navigate healthcare systems and decision-making.

Three sub-themes reflect the specific information needs of this group. The first, Condition-Specific Information Needs, relates to women’s queries about the classification and severity of their prolapse, as well as how the condition interacts with other aspects of health and bodily function. Lifestyle Information Needs relates to concerns around safe physical activity, weight management, work, and social participation. Treatment and Investigation Information Needs covers access to care, waiting times, treatment options, eligibility, risks and benefits, and what to expect from clinical assessments. Collectively, these sub-themes demonstrate that the provision of information is not supplementary to care but central to how women understand their condition, make decisions, and advocate for themselves during their healthcare journeys.

1. Condition-Specific Information Needs

Condition-Specific Information Needs reflect women’s desire to understand the nature, progression, and implications of their prolapse diagnosis. Often, patients sought clarity on the type, stage, and severity of their prolapse, sometimes expressing confusion or uncertainty about what their diagnosis meant. Many also articulated a need for practical guidance on symptom management, particularly in relation to pain, as well as sexual, urinary, and bowel dysfunction.

*‘Is it a cystocele or rectocele or both?’*

*(age 33)*

*‘Advice on how sex could be less painful because of tightness and dryness’*

*(age 32)*

**Figure 3:**
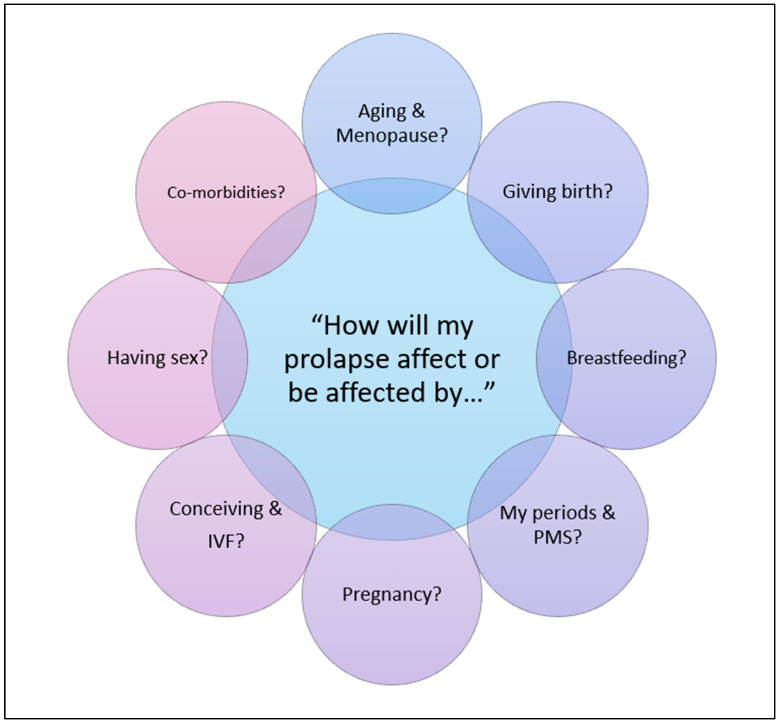
Information needs of younger women: How does prolapse relate to other health concerns?

Women frequently asked about whether their symptoms would worsen, whether prolapse could be reversed, and what they could do to facilitate this. Uncertainty about the future course of their condition, especially in relation to aging and menopause, was also common (Figure 3).

*‘What has caused this prolapse? i.e., can I change something I am doing to stop it getting any worse?’*

*(age 25)*

*‘Will my prolapse worsen with menopause?’*

*(age 34)*

Critically, women questioned how prolapse might interact with other health concerns (Figure 3). For many, the most pressing concerns related to fertility, future pregnancy, and birth experiences, including the potential impact of prolapse on conception, IVF, and delivery options. Others were concerned about issues such as menstruation, breastfeeding, premenstrual syndrome (PMS), and concurrent gynecological conditions.

*‘To know how to manage periods following changes to cervix and vaginal wall’*

*(age 33)*

*‘Will future pregnancies affect my prolapse? Will I be able to have another natural birth?’*

*(age 26)*

2. Lifestyle Information Needs

Lifestyle Information Needs represent women’s concerns about how to manage prolapse while maintaining everyday activities. Although some questions related to functioning at work or engaging in social activity, the most prevalent queries focused on exercise and exertion. Many women sought reassurance about whether they could resume physical activity and, if so, which types of exercise were safe to do. A recurring concern was a fear of exacerbating their condition, with women asking which activities to avoid and how to exercise without worsening symptoms, such as incontinence or pelvic pressure. Some also wanted to understand whether lifestyle change, particularly exercise and weight management, could positively influence their symptoms or disease progression. This reflects a broader uncertainty around how to engage in activities safely while living with prolapse, and a need for clear, evidence-based guidance tailored to the functional needs of this group.

*‘What can I do to help myself (I know I need to lose weight)?’*

*(age 38)*

*‘Is swimming or any activity like that bad for the prolapse? Could it lead to infection?’*

*(age 40)*

3. Treatment and Investigations

This sub-theme reflects women’s needs for detailed, reliable information regarding diagnostic pathways and treatment options, reflecting ongoing uncertainty about clinical processes and care trajectories. These information needs are clustered around four key areas and emphasize the importance of accessible, transparent, and individualized discussions about treatment and care, tailored to women’s specific life circumstances, health priorities, and long-term wellbeing.

  i. Treatment Eligibility and Options

Women frequently questioned whether they were eligible for specific interventions, including conservative approaches, surgical procedures, and adjunct therapies such as psychological counselling. Uncertainty around when surgery would be considered, and whether it was mandatory to try conservative options first, reflected confusion about clinical decision-making processes and the availability of individualized care.

*‘What are the treatment options available’*

*(age 33)*

*‘At what point would surgical management be considered for my cystocele?’*

*(age 40)*

  ii. Diagnostic and Intervention Processes

Many women sought greater clarity on the nature and purpose of clinical assessments, including how findings would inform treatment planning. Some requested detailed information about the investigative process and what to expect during hospital-based care, such as whether procedures would require overnight admission. There was particular interest in the types of surgical interventions offered, including the mode of surgery, and whether individual preferences and concerns would be considered in shared decision-making.

*‘Can you examine me both standing and lying and what difference does that make to prolapse grade?’*

*(age 38)*

*‘Is the operation open surgery? Don’t want laparoscopic surgery as hernia risk is higher’*

*(age 48)*

  iii. Navigation of Healthcare Systems

Queries relating to treatment access and logistics were a common topic in women’s responses. Women asked about referral timelines, treatment duration, and whether care could be accessed through the NHS or required private provision. These questions often revealed broader difficulties in navigating complex and at times opaque care pathways.

*‘Is my problem difficult? Does it need a long treatment journey?’*

*(age 33)*

*‘Can I be treated under NHS umbrella, I mean not in private clinic?’*

*(age 35)*

  iv. Expectations, Risks, and Recovery

In their responses, many women queried what outcomes to expect from treatment, both in terms of symptom relief and complications. Questions around scarring, loss of sensation, or incontinence following treatment were common, as were concerns about recovery time, pain management, time off work, and the level of support needed during convalescence. There was particular uncertainty around what post-treatment ‘success’ looks like, highlighting difficulties encountered by some women in making treatment decisions.

*‘Will I need to rest? Will my partner have to take time of work? Will I be in pain?’*

*(age 31)*

*‘What are the recovery times, and will I be continent?’*

*(age 48)*

## 4. Discussion

This study provides new evidence on the symptom burden, treatment goals, and information needs of younger women complaining of POP, a group typically underrepresented in both research and clinical guidance. Analysis was informed by the biopsychosocial model [56,57,58] and Cohen’s Framework for Women’s Health [59] to support a comprehensive evaluation of the factors that may influence this population’s experiences of health and healthcare.

Statistical analysis of ePAQ-PF scores showed that women aged ≤50 years reported significantly greater symptom severity and bother across multiple pelvic floor domains, compared with their older counterparts. These findings highlight a counterintuitive trend in which younger women experience a greater burden of prolapse-related symptoms despite being less likely to present with advanced anatomical severity. These results are consistent with existing literature [27,28], which refutes the assumption that prolapse is inherently less burdensome in younger women. While the underlying reasons are not clear from this dataset, further qualitative research is underway to explore this in more depth.

Qualitative content analysis of free-text data identified five key health-related themes, each encompassing sub-themes describing prolapse-related symptom burden and associated treatment goals. These findings support existing research on prolapse-related quality of life [7,11,12,13], revealing complex, far-reaching effects that extend beyond physical symptoms to include impacts on mobility, work, relationships, self-image, and mental health. Furthermore, the specific focus on younger women in this study revealed additional concerns unique to this group, particularly regarding reproductive health, menstruation, and early parenting experiences, which aligns with the limited but growing body of evidence in this area [6,14,18].

Treatment goals were similarly diverse, including symptom relief as well as restoration of physical function, confidence, and emotional wellbeing. Many women emphasized the need for treatment approaches sensitive to their sexual and reproductive health priorities. Their accounts also highlighted that overall wellbeing is closely linked to the quality and responsiveness of care, and that treatment needs may change over time. These findings contribute to the existing evidence base on treatment goals in pelvic floor medicine [40,41,42,43,44,45,46], offering important insights into the distinct needs of younger women. They also align with recent work by Carlin et al. [14], which identified family planning considerations as central to POP treatment decision-making and reinforced the need for comprehensive counselling and personalized treatment options prior to surgical intervention.

A sixth cross-cutting theme, Information Needs, was central to women’s ability to understand, navigate, and manage their condition. Women sought a better understanding of their diagnosis, prognosis, and symptom progression, alongside guidance on lifestyle adaptation, options, and eligibility for treatment, and what to expect from clinical assessments and recovery. Across responses, it was evident that information provision was viewed as fundamental to informed decision-making, empowerment, self-management, and meaningful engagement with treatment. These findings demonstrate significant gaps in current patient education and resources, highlighting the need for improved communication and support. They are consistent with studies by Carroll et al. [6], Mirskaya et al. [18], and Kearney et al. [80], who have emphasized the importance of providing tailored information to reproductive-aged women and have called for further research into the optimal timing, delivery, and content of educational interventions. This is particularly urgent given the absence of formal guidelines for counselling younger women with prolapse [14] and evidence that enhanced knowledge of pelvic floor disorders improves healthcare-seeking behavior, symptom management, and quality of life [52,81].

### 4.1. Strengths and Limitations

A key strength of this study is its use of a large multi-site ePAQ-PF dataset (n = 1473), enabling robust comparison of symptom severity and bother across age groups. Qualitative content analysis of a substantial sub-sample of 399 women aged ≤50 years enhances the generalizability of findings to the broader population of younger women with prolapse. The self-administered nature of ePAQ-PF may also encourage greater disclosure of sensitive issues than face-to-face interviews. While qualitative interviews may have provided greater depth and specificity, the free-text responses yielded valuable insights into women’s treatment concerns, expectations, and priorities, offering a meaningful contribution to this under-researched population. Finally, grounding the analysis in the biopsychosocial model [56,57,58] and Cohen’s Framework for Women’s Health [59] supported a holistic interpretation of women’s experiences.

Several limitations should be acknowledged. First, the lack of access to clinical records meant that women were categorized based on self-reported prolapse, which may limit accuracy. Nevertheless, both ePAQ-PF scores and free-text content strongly indicated prolapse as a primary concern. Second, defining younger women as those aged 50 and under may be viewed as a limitation, given the typical decline in fertility before this age. This threshold was chosen to include women potentially within their reproductive years, using the average age of natural menopause (approximately 51 years) as a biologically meaningful reference point [54,55]. Third, while the use of two discrete age groups could be considered a limitation, this approach was selected to align with the qualitative component of the analysis. Finally, ePAQ-PF collects limited demographic information, excluding variables such as ethnicity, socioeconomic status, and gender identity, which constrained our ability to examine differences across diverse patient groups. Future research should prioritize the collection of broader demographic data to ensure that all individuals affected by pelvic floor conditions are represented and inform inclusive service development. Additionally, studies employing purposive sampling, verified clinical diagnoses, and analyses of prolapse symptom severity and impact across age groups would further strengthen the evidence base.

### 4.2. Implications for Research and Clinical Practice

These findings have important implications for clinical practice, patient information and education, and service delivery. Recommendations were developed in collaboration with the PPI panel, whose lived experience was instrumental in ensuring the relevance and acceptability of proposed changes.

First, the qualitative findings demonstrate that prolapse has far-reaching functional, psychosocial, and reproductive impacts in younger women, which may not always be considered in objective clinical assessments. Clinicians should be aware that women with prolapse may experience a high symptom burden, regardless of anatomical severity, and require a holistic, person-centered approach to care that goes beyond condition-specific symptom management.

In addition, women frequently reported confusion about the availability and eligibility criteria for different treatment options within the NHS, as well as uncertainty around expected outcomes and what constitutes treatment ‘success.’ This underscores the importance of transparent, standardized treatment pathways that ensure women are fully informed about their options and are supported in decision-making that corresponds to their life stage, personal circumstances, and treatment goals.

Moreover, the findings revealed that many women were uncertain about their prognosis, treatment expectations, and how to engage in self-care. The PPI panel emphasized the lack of accessible, reliable health information as a key barrier to understanding and participating in care. Developing age-specific, evidence-based information resources is therefore essential. In particular, NHS websites should be reviewed and expanded to provide up-to-date, comprehensive content on topics such as menstruation, exercise, fertility, conception, pregnancy, and postnatal care in the context of prolapse.

Lastly, the PPI panel identified a need for greater public and professional awareness of prolapse. Integrating education into routine care, such as cervical screening or antenatal/postnatal appointments, could help women recognize symptoms earlier and access timely support. For those considering future pregnancies, clinicians should offer personalized counselling on potential risks, treatment options, and available support during pre-conception care and throughout the perinatal period.

To support women in making informed choices, further research might focus on the development and evaluation of age-specific shared decision-making tools and evidence-based educational resources. Co-production with patients will be essential to ensure these resources are relevant, accessible, and responsive to the specific needs and priorities of younger women living with prolapse.

## 5. Conclusions

This study offers important new insights into the lived experiences, treatment priorities, and information needs of younger women with pelvic organ prolapse. Addressing the specific needs of this population will require transparent and consistent treatment pathways, enhanced access to tailored information, and ongoing patient involvement in the development of guidelines, resources, and services.

## Figures and Tables

**Table 1:** Demographic characteristics of women aged ≤50 and >50 years self-reporting prolapse.

Variable	Aged ≤50 Years (*n* = 399)	Aged >50 Years (*n* = 1074)
Age (years)	Mean (SD): 40.9 (6.6) Range: 22–50	Mean (SD): 66.0 (9.0) Range: 51–90
Number of children	Median (IQR): 2 (2–3) Range: 0–9	Median (IQR): 2 (2–3) Range: 0–10
Body Mass Index (BMI)	Mean (SD): 27.2 (5.5) Range: 16–53	Mean (SD): 27.1 (5.1) Range: 14–66

**Table 2:** Comparison of symptom and quality of life scores between women aged ≤50 and >50 years self-reporting prolapse.

Domain Score ^1^	Aged ≤50 Years Median (IQR)	Aged >50 Years Median (IQR)	*p*-Value	Significant at *p* = 0.0025	Higher Overall Score (Median and IQR)	Effect Size (*r*)
Pain and Sensation—Urinary	11.1 (0.0–33.3)	11.1 (0.0–22.2)	0.153	No	Not significant	0.037
Voiding—Urinary	16.7 (8.3–33.3)	16.7 (8.3–33.3)	0.903	No	Not significant	0.003
Overactive—Bladder	20.0 (13.3–40.0)	26.7 (13.3–40.0)	<0.001	Yes	>50 years	0.087
Stress Urinary Incontinence	26.7 (6.7–46.7)	20.0 (0.0–33.3)	<0.001	Yes	≤50 years	0.150
QoL—Urinary	33.3 (11.1–66.7)	22.2 (11.1–55.6)	0.002	Yes	≤50 years	0.080
Irritable Bowel	26.7 (13.3–46.7)	20.0 (6.7–33.3)	<0.001	Yes	≤50 years	0.170
Constipation	22.2 (11.1–44.4)	11.1 (11.1–33.3)	0.002	Yes	≤50 years	0.079
Evacuation—Bowel	19.1 (9.5–38.1)	14.3 (0.0–23.8)	<0.001	Yes	≤50 years	0.175
Continence—Bowel	9.5 (0.0–23.8)	9.5 (0.0–23.8)	0.858	No	Not significant	0.005
QoL—Bowel	11.1 (0.0–44.4)	0.0 (0.0–33.3)	<0.001	Yes	≤50 years	0.138
Body Image	33.3 (8.3–66.7)	0.0 (0.0–25.0)	<0.001	Yes	≤50 years	0.311
Pain and Sensation—Vagina	25.0 (16.7–41.7)	16.7 (8.3–35.4)	<0.001	Yes	≤50 years	0.154
Capacity—Vagina	0.0 (0.0–11.1)	0.0 (0.0–0.0)	0.008	No	≤50 years	0.069
Prolapse	50.0 (25.0–75.0)	41.7 (16.7–66.7)	<0.001	Yes	≤50 years	0.112
QoL—Vagina	44.4 (22.2–77.8)	22.2 (11.1–66.7)	<0.001	Yes	≤50 years	0.175
Sex and Urinary	25.0 (0.0–58.3)	0.0 (0.0–33.3)	<0.001	Yes	≤50 years	0.236
Sex and Bowel	0.0 (0.0–33.3)	0.0 (0.0–0.0)	<0.001	Yes	≤50 years	0.244
Sex and Vagina	41.7 (16.7–66.7)	0.0 (0.0–41.7)	<0.001	Yes	≤50 years	0.315
Dyspareunia	26.7 (13.3–46.7)	0.0 (0.0–26.7)	<0.001	Yes	≤50 years	0.348
General Sex Life	50.0 (25.0–75.0)	16.7 (0.0–50.0)	<0.001	Yes	≤50 years	0.331

^1^ Scores range from 0 (best health status) to 100 (worst health status).

**Table 3:** Comparison of impact scores between women aged ≤50 and >50 years self-reporting prolapse.

Impact Score ^1^	Aged ≤50 Years Median (IQR)	Aged >50 Years Median (IQR)	*p*-Value	Significant at *p* = 0.0029	Higher Overall Score (Median and IQR)	Effect Size (*r*)
Pain and Sensation—Urinary	0.0 (0.0–1.0)	0.0 (0.0–1.0)	0.228	No	Not significant	0.031
Voiding—Urinary	1.0 (0.0–2.0)	1.0 (0.0–2.0)	0.009	No	Not significant	0.068
Overactive Bladder	1.0 (0.0–2.0)	1.0 (0.75–2.0)	0.027	No	Not significant	0.058
Stress Urinary Incontinence	1.0 (0.0–2.0)	1.0 (0.0–1.0)	<0.001	Yes	≤50 years	0.162
Irritable Bowel	1.0 (0.0–2.0)	1.0 (0.0–2.0)	<0.001	Yes	Equal	0.132
Constipation	1.0 (0.0–2.0)	1.0 (0.0–1.0)	<0.001	Yes	≤50 years	0.126
Evacuation—Bowel	1.0 (1.0–3.0)	1.0 (0.0–2.0)	<0.001	Yes	≤50 years	0.211
Continence—Bowel	1.0 (0.0–2.0)	1.0 (0.0–2.0)	0.047	No	Not significant	0.052
Body Image	1.0 (0.0–3.0)	0.0 (0.0–1.0)	<0.001	Yes	≤50 years	0.311
Pain and Sensation—Vagina	2.0 (1.0–3.0)	1.0 (0.0–2.0)	<0.001	Yes	≤50 years	0.236
Capacity—Vagina	0.0 (0.0–1.0)	0.0 (0.0–0.0)	0.001	Yes	≤50 years	0.084
Prolapse	2.0 (2.0–3.0)	2.0 (1.0–3.0)	<0.001	Yes	≤50 years	0.197
Sex and Urinary	1.0 (0.0–3.0)	0.0 (0.0–1.0)	<0.001	Yes	≤50 years	0.274
Sex and Bowel	0.0 (0.0–2.0)	0.0 (0.0–0.0)	<0.001	Yes	≤50 years	0.265
Sex and Vagina	2.0 (1.0–3.0)	0.0 (0.0–2.0)	<0.001	Yes	≤50 years	0.380
Dyspareunia	2.0 (1.0–3.0)	0.0 (0.0–2.0)	<0.001	Yes	≤50 years	0.379
General Sex Life	2.0 (1.0–3.0)	0.0 (0.0–2.0)	<0.001	Yes	≤50 years	0.374

^1^ Scores are based on a 4-point Likert scale ranging from 0 (‘not a problem’) to 3 (‘a serious problem’), indicating the degree of symptom-related bother.

## Data Availability

The original contributions presented in this study are included in the article. Further inquiries can be directed to the corresponding author.

## Abbreviations

The following abbreviations are used in this manuscript:

ePAQ-PF	electronic Personal Assessment Questionnaire—Pelvic Floor
POP	Pelvic organ prolapse
PPI	Patient and Public Involvement
QoL	Quality of life
NHS	National Health Service (UK)

## Appendix A

1. Symptom Burden—Key issues and illustrative quotes
2. Treatment Goals—Key issues and illustrative quotes
3. Information Needs—Key issues and illustrative quotes

**Table A1:** Symptom Burden—Key issues and illustrative quotes.

Theme	Symptom Burden	Illustrative Quote
Theme 1: Physical Health	Physical symptoms	*‘Prolapse is hanging out all the time’ (age 39)* *‘Constant irritation in my vagina’ (age 40)* *‘Pain, bloating and continuous discomfort in my abdomen and back’ (age 47)* *‘Prolapse in my bottom is affecting me when I go to the toilet’ (age 36)* *‘Vaginal prolapse and dragging heaviness in abdomen’ (age 39)* *‘Wind and bowel urgency and incontinence. Bladder incontinence’ (age 43)* *‘Bowel problems, having to keep taking laxatives as I have been doing for many years’ (age 48)* *‘The pain makes me feel sick often’ (age 39)*
Theme 2: Functionality	Functional impact on daily life	*‘Avoiding exercise for fear of long-term damage/incontinence’ (age 36)* *‘Ring pessary inserted in May. Not helped, various sizes tried as unable to walk a short distance even with the pessary’ (age 45* *)* *‘My prolapse is always out of my vagina. It’s very uncomfortable and sore and stings a lot of the time when I’m working’ (age 50)* *‘I kept having excruciating pain in my tummy and vaginal area. I took myself to hospital and a gynaecologist said I had a prolapse of the womb, as well as loose skin which hangs down, causing more pain/infections/embarrassment and [difficulties in] the enjoyment of a sex life/social activities/day to day housework/exercise and anything which involves moving around’ (age 33)*
Theme 3: Psychosocial & Emotional Wellbeing	Impact to psychosocial & emotional wellbeing	*‘I’m really depressed about my vagina and how it makes me feel.’ (age 23)* *‘I have lost my sex life and nearly my marriage after the depression this has caused.’ (age 46)* *‘Feel more attractive again […] My mental health is not good due to this’ (age 42)* *‘I have a toddler and 10-year-old, and I can’t even be the mum they deserve’ (age 32)*
Theme 4: Reproductive & Sexual Health	Impact to sexual function & satisfaction	*‘Currently not having sex due to prolapse’ (age 41)* *‘Swelling of vagina after sex’ (age 26)* *‘This is serious for me, I can’t meet anyone or be with anyone, because sex is important and I want to have sex with someone and feel sexy, but I just can’t’ (age 23)* *‘Have to empty my bowel prior to having sex for fear of leakage during sex’ (age 45)*
	Pregnancy & childbirth concerns	*‘Has my child done damage which has gone unnoticed?’ (age 31)* *‘After birth of 2nd child vagina opening is too wide due to stitches coming undone’ (age 39)* *‘First daughter ventouse (now 9) and second daughter (now 5 years old), forceps which caused the problems’ (age 40)*
	Menstrual/cyclical health concerns	*‘Prolapse bulging, inability to keep tampon in’ (age 33)* *‘To discuss why using tampons leads to left-sided moderate to severe lower abdominal pain’ (age 41)* *‘My bladder issues are very related to my cycle. What holistically can be done?’ (age 41)*
Theme 5: Healthcare Journeys	Ongoing treatment concerns	*‘Vaginal ring pessary inserted at appointment as moderate prolapse […] Really suffering with pelvic pain and back ache and anus pressure’ (age 35)* *‘‘Why can’t I wee when I don’t have antibiotics, yet I can only when I am on them’ (age 32)* *‘I had a Rectopexy […] I have now developed superficial dyspareunia to the extent that penetration is completely impossible’ (age 50)*
	Future treatment concerns	*‘Because I also have a slight rectocele, will my cystocele repair make the rectocele more of a problem?’ (age 49)* *‘I am only 45 and I have concerns about non-surgical treatment that has to be repeated regularly’ (age 45)*
	Perceptions of care	*‘I was very apprehensive before attending as it’s a very personal issue. Everyone I met was supportive and understanding and put me at my ease. I feel I received appropriate advice, and the proposed treatment plan is as I hoped.’ (age 27)* *‘[Healthcare professional] on the ward was very kind and matter of fact which made me feel more comfortable about what is an uncomfortable issue.’ (age 43)* *‘Treatment been poor, feel consultant covering up. The gynae department is known not to be up to standard […] there are a lot of problems, and they have let me down badly.’ (age 49)* *‘My initial concerns post-op were dismissed which resulted in emergency surgery. Supposed to have follow up or response* via *PALS. Not had post-op meeting with head of gynaecology’ (age 46)* *‘I feel that I have been forced to suffer and put at great physical risk by red tape that delayed the whole process’ (age 41)*

**Table A2:** Treatment Goals—Key issues and illustrative quotes.

Theme	Treatment Goal	Illustrative Quote
Theme 1: Physical Health	Symptom reduction & improvement - prolapse, urinary, bowel, vaginal and gastro-intestinal symptoms	*‘Not to have to wear pads and sneeze, cough, laugh without leaking.’ (age 30)* *‘To sort my prolapse so that I can empty my bowels like a normal person.’ (age 50)* *‘No dragging or feeling like everything is falling out down below.’ (age 36)* *‘No bulging into my vagina as it feels horrid.’ (age 34)* *‘Ease bloating.’ (age 47)* *‘Get rid of prolapse so that I can open my bowels without having to support the bowel vaginal wall’ (age 45)*
	Symptom reduction & improvement - pain/discomfort	*‘Help ease constant discomfort.’ (age 34)* *‘I hope to have less abdominal pain and discomfort because I suffer every day from abdominal issues.’ (age 32)* *‘Improve pain management of prolapse in terms of general dragging pain, plus soreness and abrasion.’ (age 37)*
	Management of other gynaecological Health concerns	*‘Polycystic ovaries gone.’ (age 43)* *‘Sort prolapse and fibroids to see what’s causing abdominal pain.’ (age 50)*
Theme 2: Functionality	Functional improvement - work life	*‘To be able to work […] and live my life without my uterus hanging in my knickers.’ (age 41)* *‘[To] feel confident in work and social circumstances.’ (age 50)* *‘Being able to do my job without having to worry about prolapse and leakage.’ (age 49)*
	Functional improvement - ability to engage in daily activities	*‘Housework/childcare without being limited by vaginal prolapse.’ (age 40)* *‘Resume regular day to day activities.’ (age 38)*
	Functional improvement - mobility & exercise	*‘Be able to do exercise I enjoy.’ (age 30)* *‘To be able to exercise without worry of further damage.’ (age 49)* *‘To run around after my children without fear of leaking.’ (age 30)* *‘I need to be able to run and play with my children.’* *‘Help returning to a normal life,* i.e., *[…] walking without pain’ (age 28)* *‘To find a pessary that works so I can work out again and lift children whilst keeping pressure off pelvic floor’ (age 38)*
Theme 3: Psychosocial & Emotional Wellbeing	Stronger social and family relationships	*‘Be able to spend more time with family and friends.’ (age 28)* *‘Self-confidence with my husband.’ (age 36)* *‘Be the wife and mum I felt I was failing so miserably at.’ (age 35)*
	Improved mental health and positive self-image	*‘Wish to achieve confidence, self-esteem.’ (age 26)* *‘I’d like a normal looking vagina […] I’d like my confidence back.’ (age 39)* *‘[To] not feel so disgusting all the time.’ (age 32)* *‘To improve my life, to help me socialise and decrease depression from isolation’ (age 48)* *‘Correction of prolapse […] so I can enjoy dancing (nights out) and confidence, no longer being embarrassed […] stop taking antidepressants’ (age 42)*
	Enhanced quality of life	*‘To get back to normal everyday life.’ (age 30)* *‘To please give me my life back.’ (age 46)* *‘Better quality of life. Increase in health.’ (age 49)* *‘Be able to live a more normal life’ (age 28)*
Theme 4: Reproductive & Sexual Health	Restoration of sexual function and satisfaction	*‘Improve vaginal sensation during sex.’ (age 36)* *‘To be able to have a sexual relationship.’ (age 46)* *‘Reduce pain/discomfort during sexual intercourse & regain more enjoyment of sex.’ (age 36)* *‘To be able to have a sex life without embarrassment by being tighter and looking normal externally’ (age 39)*
	Support relating to reproductive health	*‘We would like another child safely.’ (age 37)* *‘[To] be able to get pregnant in the near future.’ (age 35)* *‘Since I have given birth, my condition has gotten worse, and I have not received any further treatment’ (age 26)* *‘Relief from pain of episiotomy scar tissue. Possible refashioning which I was due to have [date]. Regain control over my pelvic floor’ (age 38)* *‘Do I have non-surgical options as considering one more baby at some point?’ (age 36)* *‘I have no interest in having children biologically, therefore am I eligible for prolapse surgery?’ (age 28)*
	Support relating to contraception	*‘Would the coil help?’ (age 41)* *‘Sterilisation to prevent further pregnancy.’ (age 27)* *‘Can I be sterilised and will it help?’ (age 42)*
	Improved menstruation experiences	*‘To be able to comfortably wear a tampon.’ (age 30)* *‘Reduce heavy feeling in vagina when due a period.’ (age 47)* *‘Irregular bleeding to be resolved.’ (age 30)* *‘Strengthening pelvic floor to use a menstrual cup again’ (age 32)* *‘How can I improve my PMS and heavy periods?’ (age 39)* *‘To be able to wear tampons without them falling out’ (age 40)*
Theme 5: Healthcare Journeys	Exploring & understanding treatment options	*‘To be timely offered the relevant intervention aimed to resolve the problem (prolapse).’ (age 46)* *‘Discuss everything including past surgeries diagnosis and prognosis.’ (age 43)* *‘Consider different pessaries and if oestrogen cream is an option.’ (age 37)* *‘How can I go about trying other pessaries (ring pessary has not worked for me)’ (age 38)* *‘I don’t want that mesh thing that causes horrible pain and infection!’ (age 49)* *‘Can you please do this operation so I can start living again as for 2 years I have had hell.’ (age 46)*

**Table A3:** Information Needs—Key issues and illustrative quotes.

Theme	Information Needs	Illustrative Quote
Theme 6: Information Needs	Condition-specific information needs	*‘Is it a cystocele or rectocele or both?’ (age 33)* *‘How severe are my symptoms?’ (age 38)* *‘Advice on how sex could be less painful because of tightness and dryness’ (age 32)* *‘Is the bulge from my rectum into my vagina during bowel movements likely to get worse?’ (age 40)* *‘What has caused this prolapse?* i.e., *can I change something I am doing to stop it getting any worse?’ (age 25)* *‘Will my prolapse worsen with menopause?’ (age 34)* *‘To know how to manage periods following changes to cervix and vaginal wall’ (age 33)* *‘Will future pregnancies affect my prolapse? Will I be able to have another natural birth?’ (age 26)* *‘Will it interfere with future IVF treatment?’ (age 33)* *‘Can this affect me being able to conceive again?’ (age 40)*
	Lifestyle information needs	*‘What can I do to help myself (I know I need to lose weight)?’ (age 38)* *‘Can I improve my urge incontinence when exercising?’ (age 39)* *‘What exercise can I do now without making my condition worse? (age 41)* *‘Is swimming or any activity like that bad for the prolapse? Could it lead to infection?’ (age 40* *)*
	Treatment eligibility & options	*‘What are the treatment options available’ (age 33)* *‘At what point would surgical management be considered for my cystocele?’ (age 40)* *‘Psychosexual counselling, is this still available?’ (age 36)* *‘Can you opt for surgery straight out or do you have to try the other options first’ (age 49)*
	Diagnostic & intervention processes	*‘Can you examine me both standing and lying and what difference does that make to prolapse grade?’ (age 38)* *‘Will more be done to try and find the reason behind my chronic stomach pains and discomfort?’ (age 32)* *‘Is it day surgery or overnight stay if having operation?’ (age 42)* *‘Is the operation open surgery? Don’t want laparoscopic surgery as hernia risk is higher’ (age 48)*
	Navigation of healthcare systems	*‘What is the next process and how long is the wait’ (age 39)* *‘Time frames for potential solutions? how long are the treatments?’ (age 30)* *‘Is my problem difficult? Does it need a long treatment journey?’ (age 33)* *‘Can I be treated under NHS umbrella, I mean not in private clinic?’ (age 35)*
	Expectations, risks & recovery	*‘Will I need to rest? Will my partner have to take time of work? Will I be on pain?’ (age 31)* *‘What are the recovery times, and will I be continent?’ (age 48)* *‘Will surgery put me at increased risk of scarring inside my vagina so risking reduced sensation’ (age 37)*
